# A Low-Intensity Mobile Health Intervention With and Without Health Counseling for Persons With Type 2 Diabetes, Part 1: Baseline and Short-Term Results From a Randomized Controlled Trial in the Norwegian Part of RENEWING HEALTH

**DOI:** 10.2196/mhealth.3535

**Published:** 2014-12-11

**Authors:** Astrid Torbjørnsen, Anne Karen Jenum, Milada Cvancarova Småstuen, Eirik Årsand, Heidi Holmen, Astrid Klopstad Wahl, Lis Ribu

**Affiliations:** ^1^Department of NursingFaculty of Health SciencesOslo and Akershus University College of Applied SciencesOsloNorway; ^2^Institute of Health and SocietyDepartment of General Practice, Faculty of MedicineUniversity of OsloOsloNorway; ^3^Department of Occupational Therapy, Prosthetics and OrthoticsFaculty of Health SciencesOslo and Akershus University College of Applied SciencesOsloNorway; ^4^Department of NursingFaculty of Health SciencesOslo and Akershus University College of Applied SciencesOSLONorway; ^5^Norwegian Centre for Integrated Care and TelemedicineUniversity Hospital of North NorwayTromsøNorway; ^6^Institute of Health and SocietyDepartment of Health Sciences, Faculty of MedicineUniversity of OsloOsloNorway

**Keywords:** self-care, quality of life, diabetes mellitus, type 2, randomized controlled trials, telemedicine, mHealth, mobile apps, counseling, complex intervention, life style

## Abstract

**Background:**

Self-management support for people with type 2 diabetes is essential in diabetes care. Thus, mobile health technology with or without low-intensity theory-based health counseling could become an important tool for promoting self-management.

**Objectives:**

The aim was to evaluate whether the introduction of technology-supported self-management using the Few Touch Application (FTA) diabetes diary with or without health counseling improved glycated hemoglobin (HbA_1c_) levels, self-management, behavioral change, and health-related quality of life, and to describe the sociodemographic, clinical, and lifestyle characteristics of the participants after 4 months.

**Methods:**

A 3-armed randomized controlled trial was conducted in Norway during 2011-2013. In the 2 intervention groups, participants were given a mobile phone for 1 year, which provided access to the FTA diary, a self-help tool that recorded 5 elements: blood glucose, food habits, physical activity, personal goal setting, and a look-up system for diabetes information. One of the intervention groups was also offered theory-based health counseling with a specialist diabetes nurse by telephone for 4 months from baseline. Both intervention groups and the control group were provided usual care according to the national guidelines. Adults with type 2 diabetes and HbA_1c_ ≥7.1% were included (N=151). There were 3 assessment points: baseline, 4 months, and 1 year. We report the short-term findings after 4 months. HbA_1c_ was the primary outcome and the secondary outcomes were self-management (Health Education Impact Questionnaire, heiQ), behavioral change (diet and physical activity), and health-related quality of life (SF-36 questionnaire). The data were analyzed using univariate methods (ANOVA), multivariate linear, and logistic regression.

**Results:**

Data were analyzed from 124 individuals (attrition rate was 18%). The groups were well balanced at baseline. There were no differences in HbA_1c_ between groups after 4 months, but there was a decline in all groups. There were changes in self-management measured using the health service navigation item in the heiQ, with improvements in the FTA group compared to the control group (*P*=.01) and in the FTA with health counseling group compared with both other groups (*P*=.04). This may indicate an improvement in the ability of patients to communicate health needs to their health care providers. Furthermore, the FTA group reported higher scores for skill and technique acquisition at relieving symptoms compared to the control group (*P*=.02). There were no significant changes in any of the domains of the SF-36.

**Conclusions:**

The primary outcome, HbA_1c_, did not differ between groups after 4 months. Both of the intervention groups had significantly better scores than the control group for health service navigation and the FTA group also exhibited improved skill and technique acquisition.

## Introduction

People with type 2 diabetes have an increased risk of cardiovascular morbidity and mortality, but the multifactorial risk can be reduced by changes in diet, exercise, and education often combined with antihypertensives, statins, and oral glucose-lowering agents or insulin-lowering drugs [1-3]. However, type 2 diabetes is a complex disease for the individual and clinicians [4]. Furthermore, severe comorbidity may decrease the capacity for self-management and patients with a macrovascular comorbidity, such as heart failure, or other diseases not related to the diabetes (eg, depression and chronic pain) may place a lower priority on their diabetes treatment [5]. Moreover, the co-occurrence of multiple diseases is associated with obesity [6] and weight loss through behavioral change may be an essential part of the treatment [7], although findings regarding the benefits of weight loss are inconclusive [8,9].

Intensive long-term interventions related to lifestyle and obesity in patients with type 2 diabetes have achieved some effects on weight loss and improved glycemic control, but these were not enduring [10]. The treatment is also more complex when type 2 diabetes is of longer duration. Due to costly treatment, it may become necessary to differentiate between those in need of a low- or high-intensity intervention, thereby offering the patients the lowest level of effective management [11] and reducing the costs. This approach is in accordance with the Norwegian Coordination Reform, which aims to transfer treatment services from hospitals to local centers in the municipalities [12]. At present, most patients with chronic diseases are treated in primary care where they are educated to improve their self-management, which is an important activity for the successful attainment of personal health goals, and to communicate with health professionals [13]. Furthermore, the development of self-management support is recommended by international guidelines because it has also been shown to have an effect on glycemic control [14,15].

Computer-based solutions may support self-management in everyday life and research shows that mobile health tools in particular may improve glycemic control, although the findings are inconclusive [16-18]. Furthermore, few telemedicine studies have detected effects on cognitive, behavioral, or emotional outcomes [17], and few studies have measured self-management using appropriate questionnaires. Some interventions combine self-monitoring with professional support, which is based primarily on the monitoring of results by health care providers, with subsequent counseling and advice [18-22]. More research is needed in this area to determine the effects on both clinical outcomes and self-management, and to assess the benefit of providing health counseling to support patients in the implementation and maintenance of the necessary behaviors required to manage their diabetes [15].

The European Union collaborative project REgioNs of Europe WorkING together for HEALTH (RENEWING HEALTH) was set up to evaluate innovative telemedicine tools on a large scale using a specially designed framework, the Model for the Assessment of Telemedicine (MAST) [23]. The present study is from the Norwegian part of the RENEWING HEALTH network. Results from the 4-month intensified part of a 1-year intervention are presented in the present paper.

The primary aim of this paper was to assess whether the use of a mobile health self-management intervention, the Few Touch Application (FTA) diabetes diary [24], with and without a theory-based health counseling intervention, was superior to usual care in terms of glycated hemoglobin A_1c_ (HbA_1c_) levels, self-management, behavioral change (diet and physical activity), and health-related quality of life after 4 months. Further, the secondary aim was to describe sociodemographic, clinical, and lifestyle characteristics of persons volunteering to participate in such a lifestyle intervention.

## Methods

### Study Design

This study was a block randomized controlled trial (RCT) [25] with 3 parallel groups: 1 control group and 2 intervention groups using the FTA diary during the 1-year study in which 1 of the 2 groups received a strengthened intervention with health counseling. The groups are described in detail in the study protocol [26]. We had a longitudinal design with 3 assessment points: baseline, after 4 months, and after 1-year follow-up. Further, the patients’ registrations in the FTA diary were recorded continuously and transferred securely to a server for research purposes.

### Participants

We used broad eligibility criteria: age ≥18 years, diagnosed with type 2 diabetes a minimum of 3 months before inclusion, HbA_1c_ ≥7.1%, able to use the FTA system, and capable of understanding and completing the questionnaires. The exclusion criteria were mental or physical conditions that interfered with the protocol [26]. HbA_1c_ measurements needed to be available to the researchers within a 1-month window (ie, 2 weeks before or after randomization) to control the eligibility criteria [27]. Participants were recruited to the study by several routes. Firstly, through general practitioners who accepted an invitation by letter after being supplied with standard information about the protocol. Secondly, at educational “diabetes start courses” which were arranged by the health care specialist for patients newly diagnosed with diabetes, and from local public health clinics in the municipalities. Finally, a few participants were recruited through media advertising. People who stated their willingness to participate were given a letter that contained a brief summary of the study and an invitation to obtain more in-depth information at start-up group meetings arranged by the research team, each of which included a maximum of 10 participants. The participants were also allowed one-to-one meetings if group meetings were not feasible for practical reasons. The participants were randomized after they signed the informed consent form.

### Study Setting and Data Collection

Participants were from the Northern and Southeastern part of Norway because the project originated from research teams in these regions and the inclusion of participants was conducted in local start-up group meetings in the regions.

The recruitment period lasted from March 2011 to October 2012. The measurement points were at baseline, after 4 months, and after 1 year. The short-term follow-up was performed between August 2011 and January 2013.

After 4 months, all the participants were invited to attend the first follow-up meeting to complete the questionnaires. They were also asked to visit their general practitioner for measurement of their HbA_1c_ levels and collection of data from their medical records. Preferably, the general practitioners completed the patients’ case record form at the same time as the questionnaires (±14 days) and returned them to the researchers in a prepaid addressed envelope. Participants who could not attend the follow-up meeting were sent the questionnaires by mail to their postal address with a prepaid addressed envelope to return them to the study center.

### Randomization

We used a computer-generated block randomization system, which was developed and administered by the Unit of Applied Clinical Research, Institute of Cancer Research and Molecular Medicine, Norwegian University of Science and Technology, Trondheim, Norway, to ensure a good balance between the numbers and confounding factors in each of the 3 groups. The blocks were small and their sizes varied. The procedure is described in detail elsewhere [26].

### Power

Power analyses were performed before recruitment to estimate the sample size required based on the HbA_1c_ level as the primary outcome. The sample size was estimated to be 34 individuals in each group with a decrease in the HbA_1c_ level of 0.35%, a significance level of 5%, a standard deviation (SD) in the outcome variable of 0.5, statistical power of 80%, and a 2-tailed significance test. To compensate for dropouts, the sample size was set to 50 in both intervention groups and 50 in the control group (total=150).

### Control Group

The control group received usual care according to the Norwegian clinical guidelines [28]; patients with type 2 diabetes are recommended to consult their general practitioner every 2-6 months and to have a more thorough consultation once a year with measurements of their blood pressure, serum lipids, glucose, HbA_1c_, weight, body mass index (BMI), etc. The treatment target for HbA_1c_ in Norway is ≤7.0% [28].

### Intervention

In addition to the usual care provided by their general practitioners, the participants randomized to the intervention arms received either the FTA diary only or the FTA diary and health counseling, which are described subsequently and in more detail in the published protocol [26].

### Few Touch Application Intervention

Both intervention groups were given a smartphone with the FTA diary for type 2 diabetes system installed. The participants were generally not able to use the app on their own smartphone because it required a specific phone model to operate properly. They were encouraged to replace their current mobile phone with the smartphone provided for the study and use it in everyday life as an ordinary mobile phone and as a diabetes diary. The smartphone provided was a HTC HD Mini based on the Windows Mobile 6.5 operating system, and the blood glucose meter was the OneTouch Ultra Easy from LifeScan. The phone and the blood glucose meter were linked using Bluetooth wireless communication so that glucose measurements were automatically transferred to the diabetes diary part of the FTA on the phone. The FTA and smartphone intervention lasted for 1 year. The FTA is a self-management tool that comprises 5 main elements accessible to the user: (1) the blood glucose data management system, (2) food habits data management system, (3) physical activity data management system, (4) personal goal-setting system, and (5) general diabetes information look-up system [24]. The blood glucose results were transferred directly from the blood glucose monitoring system to the app via Bluetooth. The diet and physical activity systems enabled an easy way of entering such data manually into the diabetes diary by the user.

### Few Touch Application With Health Counseling Intervention

In addition to the FTA intervention described previously, the participants in this group were offered health counseling with a diabetes specialist nurse for 4 months from baseline. The health counseling was based on motivational interviewing [29], the transtheoretical model [30], and a problem-solving model [11]. The nurse also supported the participants in their use of the FTA, specifically the various elements of the tool and how to take advantage of the app. The participants received 5 telephone calls from the nurse during the first 4 months, each of which lasted for an average of 20 minutes. A schedule for each conversation was developed before the study by an interdisciplinary research team [26]. In addition, the participants could contact the diabetes specialist nurse via a secured text messaging system using their smartphones when necessary [31]. The nurse responded to the messages at least twice each week. The monitoring of the sessions showed that 38 of 50 participants (76%) completed the whole program (all 5 modules), whereas 12 participants conducted 4 modules or less. Of these, 4 participants completed 4 of 5 health counseling sessions, 2 completed 3 of 5 sessions, 4 completed 2 of 5 sessions, and 2 completed 1 of 5 sessions.

### Training

Both the FTA group and the FTA with health counseling group were trained to use the mobile phone-based system at the start-up meetings, which included a demonstration of the diabetes diary [26]. They were also provided with a manual that contained instructions on the use of the smartphone, whereas the instructions for the FTA were supplied in the form of a paper-based handbook and on a universal serial bus (USB) memory stick. In addition, the consent form informed the participants about the diary and its specific procedures. A telephone support service was available to answer questions and to help the participants with technical aspects during weekdays from 9:00 to 15:00. The participants in the FTA with health counseling group were given additional training about how to send and receive secure messages to the diabetes specialist nurse.

### Measures

We used a broad evaluation based on a complex intervention framework [32] and MAST [23]. The Consolidated Standards of Reporting Trials (CONSORT) statement for reporting of RCTs [33], CONSORT for pragmatic trials [25], and the eHealth checklist [34] were used. The primary and secondary outcomes are described in Table 1, as well as the time points for the assessments. RENEWING HEALTH established a common minimum dataset of sociodemographic and clinical characteristics for all regions in the project (Table 1). Depressive symptoms were defined based on a total Center for Epidemiologic Studies Depression Scale (CES-D) score ≥16 [35]. Behavior change was measured with diet [36,37] and physical activity [38,39] questionnaires, and with the Health Education Impact Questionnaire (heiQ) [13]. Participants who reported a minimum of 60 minutes per week of moderate to vigorous activity were categorized as physically active. Detailed descriptions of the measures and the national and international validations of the measures are given in the published protocol [26]. The Diabetes Empowerment Short-Form scale [40] (described in the protocol) demonstrated a ceiling effect; thus, the data collected using this scale were not analyzed.

**Table 1:** Data collected at baseline and after 4- and 12-month follow-ups.

Measurements		Baseline	After 4 months	After 12 months
**Sociodemographic variables**				
	Demographics, marital status, education, work situation^c^	X		
**Clinical characteristics**				
	Related to disease, self-monitoring blood glucose, late complications (foot ulcer, eye)	X		
	Comorbidity^c^ (EU minimum dataset)	X		
	Smoking and alcohol habits^c^	X		X
**Self-reported questionnaires**				
	Health-related quality of life (SF-36) version 2.0 [41]^b,c^	X	X	X
	Depression (CES-D) [35]	X	X	X
	Self-management (heiQ) [13]^b^	X	X	X
	Physical activity (from HUNT) [38] and motivation (transtheoretical model) [39]^b^	X	X	X
	Diet [36,37]^b^	X	X	X
	System Usability Scale [42]^d^		X	
	Service user technology acceptability (SUTAQ)^c,d^			X
	Participation in other courses/programs during the study^e^			X
**In-depth interviews**				
	Participants’ perceptions of the intervention^d^			X
**From general practitioners’ medical records**				
	Diabetes medication	X		
	Change in medication		X	X
	Medication in general			X
	Height^c^	X		
	Weight, blood pressure, and waist circumference^c^	X		X
	HbA_1c_^a^	X	X	X
	Lipids	X		X
	Hypoglycemic events	X	X	X
	Cardiovascular complications	X		
	Use of health care, expenses^c^		X	X
	General practitioners classification of diseases			X
**Mobile user log**					
	Log data from FTA^d^	X	X	X

^a^ Primary outcome.

^b^ Secondary outcome.

^c^ EU minimum dataset.

^d^ Only the groups receiving a mobile phone (FTA and FTA with health counseling).

^e^ Such as swimming, cooking, weight reduction.

### Blood Samples and Clinical Data

Information about the HbA_1c_ level, weight, height, blood pressure, and medication were obtained from the medical records through the case record form. The HbA_1c_ level was also measured using a DCA Vantage Analyzer (Siemens) by the research team if the HbA_1c_ results were not provided by the general practitioner or were missing for other reasons (19/269, 7.1% of total cases). The blood pressure was measured according to the standardized instructions (ie, the clinicians used the correct cuff size and the patient was sitting for a 5-minute rest before 3 measurements were obtained with 1-minute intervals) and the mean of the last 2 measurements was recorded. The waist circumference was measured at the umbilical level.

### Blinding

Blinding of participants was not possible because the participants were aware of their group allocations. The general practitioners were not blinded because the participants were encouraged to discuss the progression of their glucose measurements, diet records, and activity logs with them. The assessment of the participants’ eligibility according to the inclusion criteria and the smartphone use training were performed by the research team. The researchers were part of the project team; thus, they also knew the groups to which the participants were allocated as did the technical support team.

### Statistical Analysis

The baseline sociodemographic, clinical, treatment variables, and lifestyle characteristics were expressed as counts with percentages for categorical variables or means and SDs for continuous variables. The differences in mean change from baseline to 4-month follow-up between the groups were analyzed using 1-way analyses of variance (ANOVA) for both the primary outcome (HbA_1c_) and the secondary outcomes (heiQ and SF-36). Further, change in both primary (HbA_1c_) and secondary outcomes (heiQ and SF-36) were modeled with univariate linear regression models. To correct for possible confounding effects, we adjusted for age, gender, education, comorbidity, work situation, BMI, depression, and regions from different parts of Norway using multiple linear regression. For baseline measurements, all 3 groups were compared using the Kruskal-Wallis test.

Data that were not available were considered missing and the results were based on the intention-to-treat approach. The trend in the use of the app was described with number of glucose measurements and other keystrokes in the app. *P* values <.05 were considered statistically significant. All tests were 2-sided. The analyses were performed using SPSS version 21 (IBM Corp, Armonk, NY, USA).

### Ethics and Safety

The study was approved by the Regional Committee for Medical and Health Research Ethics. All participants gave their written informed consent before study start. The ethical guidelines and rules were followed with the intention to do well and prevent harm or risks.

The participants’ entries in the FTA diabetes diary app were recorded continuously and transferred to a secure server at 1 of the study sites (Tromsø). A comprehensive risk analysis of the technology was performed before the start of the study to ensure that privacy and security issues were addressed in an appropriate manner and the data were kept at the responsible research institutions [26]. Through the informed consent form, participants were made aware of the possibility of hypoglycemia related to behavioral change and they were informed to contact their general practitioner according to their instructions.

## Results

### Overview

**Figure 1:**
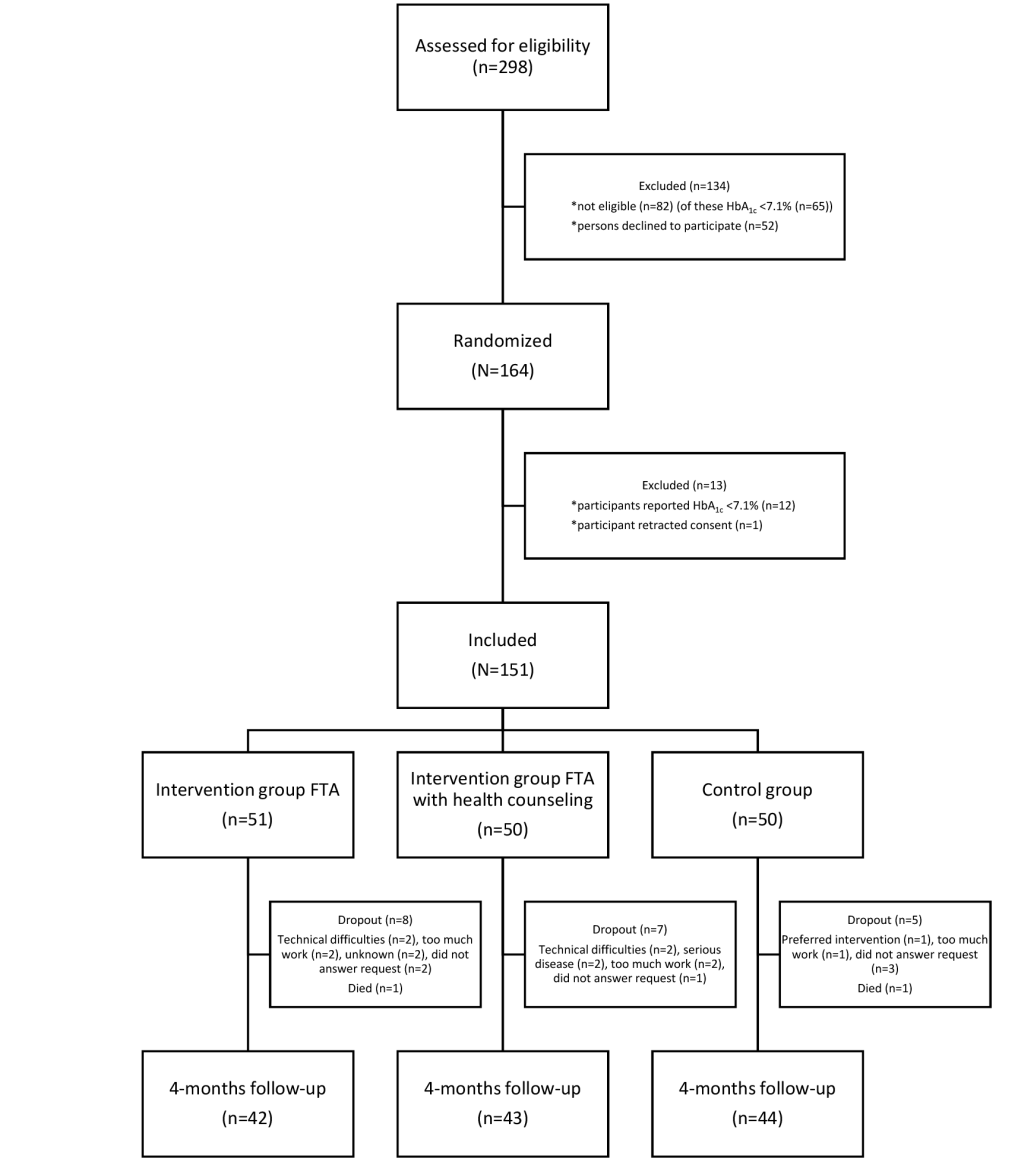
Flow diagram showing the design of the study.

In total, 298 individuals were assessed for eligibility (Figure 1), 65 of which were excluded because of HbA_1c_ levels <7.1%, 17 were not eligible due to other reasons, and 52 declined to participate. In total, 164 participants were randomized of which 151 were included in the study because 12 participants had HbA_1c_ <7.1% at the time of inclusion and 1 retracted consent.

### Baseline Characteristics of the Groups

There were no statistically significant differences between the groups in terms of the baseline variables, except for rheumatism and depressive symptoms (Table 2). Significantly more participants in the FTA group had rheumatism compared with both of the other groups (n=11, 4, and 3 in the FTA, FTA with health counseling, and control groups, respectively, *P*=.03). More individuals had depressive symptoms (a CES-D score ≥16) in the control group (n=17) compared with the FTA group (n=10) and the FTA with health counseling group (n=7, *P*=.045).

Of the 151 participants, the mean age was 57 years (SD 12), 62 (41.1%) of participants were women, and 83 (55.0%) had less than 12 years of education. The mean HbA_1c_ was 8.2% (SD 1.1) or 66 mmol/mol (SD 12), the mean BMI was 31.7 kg/m^2^ (SD 6.0), and 58.1% (75/129) were obese [43]. Only 9 of 131 participants (6.9%) did not receive glucose-lowering medication. In total, almost half of the participants (72/151, 48%) reported 2 or more comorbidities and 36 of 151 (23.8%) reported heart disease.

**Table 2:** Baseline characteristics of the control group and the 2 intervention groups.

Variables			Intervention groups		Control group (n=50)
			FTA (n=51)	FTA with health counseling (n=50)	
**Sociodemographic characteristics**					
	Age (years), mean (SD)		58.6 (11.8)	57.4 (12.1)	55.9 (12.2)
	Gender (female), n (%)		17 (33)	25 (50)	20 (40)
	Educational background <12 years, n (%)		26 (51)	26 (52)	31 (62)
**Clinical characteristics**					
	**HbA**_**1c**_ **(%), mean (SD)**		8.1 (1.1)	8.2 (1.1)	8.3 (1.2)
		HbA_1c_ (%),median (range)	7.8 (7.1-12.4)	7.9 (7.1-11.3)	7.9 (7.1-11.6)
		HbA_1c_ (mmol/mol), mean (SD)	65 (12.1)	66 (12.2)	67 (12.7)
	Comorbidity (≥2), n (%)		28 (55)	22 (44)	22 (44)
	BMI (kg/m^2^), mean (SD)		32.4 (6.5)	30.7 (5.6)	32.0 (6.0)
	**BMI range, n (%)**				
		Normal (18.50-24.99)	2 (4)	7 (16)	4 (10)
		Preobese (25.00-29.99)	17 (38)	13 (30)	11 (28)
		Obese class I (30.00-34.99)	13 (29)	14 (32)	15 (38)
		Obese class II (35.00-39.99)	8 (18)	6 (14)	6 (15)
		Obese class III (≥40)	5 (11)	4 (9)	4 (10)
		Missing data, n	6	6	10
	**Weight (kg), mean (SD)**		98 (23)	91 (20)	96 (25)
		Missing data, n	6	4	9
	**Height (cm), mean, (SD)**		173 (10)	171 (10)	172 (11)
		Missing data, n	6	6	10
	**Blood pressure (mm Hg), mean (SD)**				
		Systolic	136 (16.9)	132 (13.7)	134 (14.5)
		Diastolic	81 (8.2)	79 (8.6)	82 (9.4)
		Missing data, n	8	7	16
	**Duration of diabetes (years), mean (SD)**		11.2 (7.3)	9.6 (8.4)	9.4 (5.5)
		Missing data, n	3	5	5
**Treatment variables**					
	**Glucose-lowering medication, n (%)**				
		No medication	3 (7)	2 (4)	4 (11)
		Only oral agents	20 (44)	27 (57)	16 (42)
		Only injections	9 (20)	7 (15)	3 (8)
		Combination oral/injections	14 (30)	11 (23)	15 (40)
		Missing data, n	5	3	13
	Self-monitoring blood glucose, n (%)		48 (94)	45 (90)	49 (98)
**Lifestyle characteristics**					
	Smoking (yes), n (%)		5 (10)	12 (24)	7 (14)
	**Physical activity, n (%)**				
		Little or not engaged in physical activity	31 (63)	34 (68)	33 (66)
		Some to very engaged in physical activity	18 (37)	16 (32)	17 (34)
		Missing data, n	2	0	0

### Characteristics in Responders Versus Nonresponders

When comparing distribution of variables at baseline and at 4 months in responders versus nonresponders, there were no significant differences between the groups. Hence, our analyses of dropouts vs nondropouts indicated that attrition did not change the distribution between the groups at baseline (Table 3).

**Table 3:** Differences between responders and nonresponders at 4 months.

Variables		Responders at 4 months (n=118)	Nonresponders^a^ at 4 months (n=33)	*P*
Age (years), mean (SD)		57.9 (10.7)	55.3 (15.9)	.52^b^
Gender (female), n (%)		47 (39.8)	15 (46)	.56^c^
Education <12 years, n (%)		68 (57.6)	15 (46)	.21^c^
HbA_1c_ (%), mean (SD)		8.2 (1.1)	8.2 (1.1)	.74^b^
**BMI (kg/m^2^), mean (SD)**		31 (6.0)	34 (5.9)	.09^b^
	Missing data (BMI), n	7	15	
Comorbidities ≥2, n (%)		58 (49.2)	14 (42)	.49^c^
**Diabetes duration (years), mean (SD)**		10 (7.0)	9 (7.8)	.20^b^
	Missing data (diabetes duration), n	9	4	

^a^ Nonresponders (those without HbA_1c_ at 4 months).

^b^ Between-group differences tested with Mann-Whitney test.

^c^ Between-group differences tested with chi-square test.

### Primary Outcomes and Estimations

In total, 118/151 (78.2%) participants provided HbA_1c_ data at 4 months. There were no statistically significant differences in HbA_1c_ level changes from baseline between the 3 groups (*P*=.65) after 4 months (Table 4). Adjustments for age, gender, and education did not affect the estimates.

The mean HbA_1c_ level declined in all groups: –0.41 (95% CI –0.71 to –0.11) in the FTA with health counseling group, –0.23 (95% CI –0.47 to 0.01) in the FTA group, and –0.39 (95% CI –0.75 to –0.03) in the control group.

**Table 4:** Changes in HbA_1c_ between baseline and 4 months.

Groups		Baseline		4 months		Mean change	
Intervention		n	Mean (95% CI)	n	Mean (95% CI)	n	Mean (95% CI)
	FTA	51	8.1 (7.8, 8.4)	40	7.8 (7.5, 8.0)	40	–0.23 (–0.47, 0.01)
	FTA with health counseling	50	8.2 (7.9, 8.5)	39	7.8 (7.4, 8.2)	39	–0.41 (–0.71, –0.11)
Control		50	8.3 (8.0, 8.6)	39	8.0 (7.6, 8.4)	39	–0.39 (–0.75, –0.03)

### Secondary Outcomes

We obtained data from 124/151 (82.1%) participants who provided self-reported data at 4 months. We found that there was significantly improved self-management between baseline and 4-month follow-up with respect to 2 heiQ domains for at least 1 intervention group compared to the control group (Table 5). The participants in the FTA group reported significantly higher scores than the control group (*P*=.01) for health service navigation indicating an improved ability to discuss their health needs with their provider. Moreover, the FTA with health counseling group reported significantly higher scores than both the control group and the FTA group (*P*=.04) also after the scores were adjusted for age, gender, and education level to account for possible confounders (Table 6).

For the skill and technique acquisition domain, which indicates that the participants possess the skills and techniques required to relieve symptoms and manage health challenges, the FTA group reported significantly higher scores than the control group (*P*=.02) after adjusting for age, gender, and education level in the linear regression analyses. However, there were no differences between the FTA with health counseling group and the other 2 groups (*P*=.11). The difference between the FTA group and the control group was also found after adjusting for age, gender, and education level.

We fitted linear regression models for the health service navigation domain and the skill and technique acquisition domain and the following explanatory variables: duration of diabetes, comorbidity, work situation, BMI, depression, and regions from different parts of Norway. None of these explanatory variables were statistically significant.

There were no statistically significant differences in the changes between baseline and 4-month follow-up for health-related quality of life (SF-36) within or between the 3 groups or for changes in diet and physical activity (results not shown).

**Figure 2:**
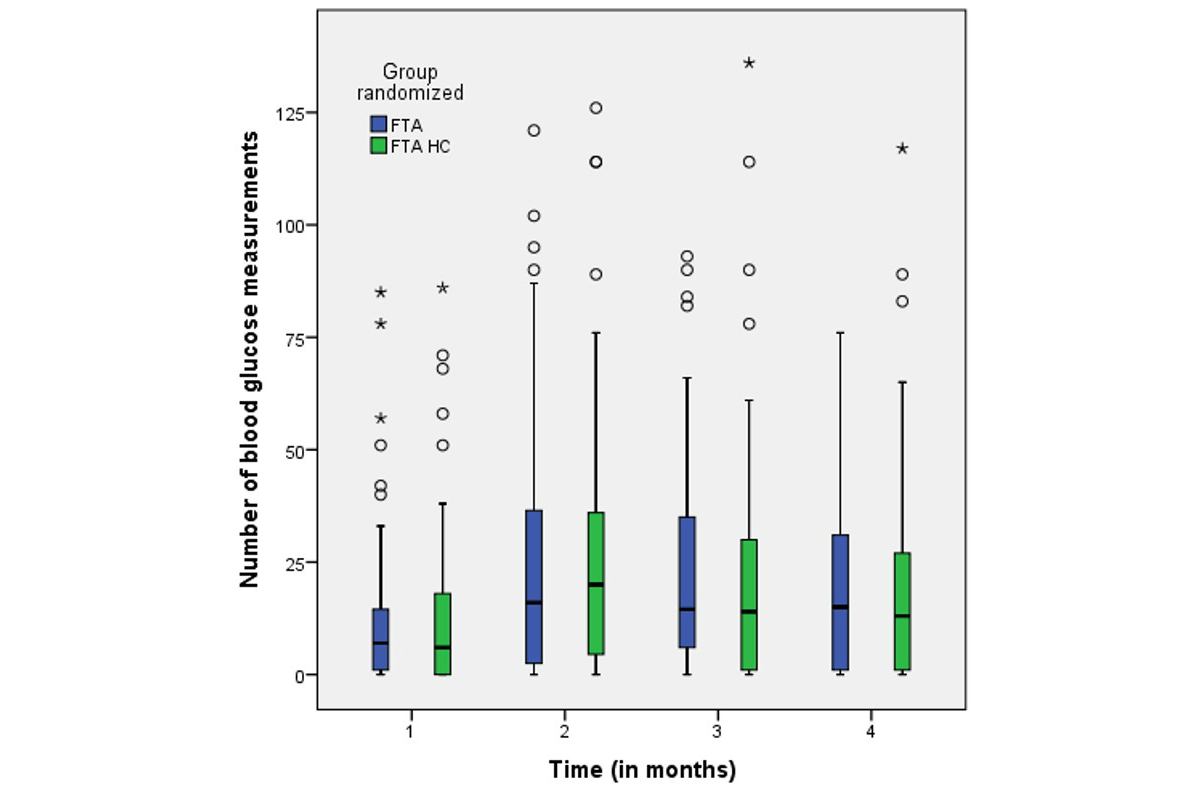
Number of blood glucose measurements during the first 4 months for the 2 intervention groups: Few Touch Application (FTA) and FTA with health counseling (HC). Time 1 (baseline): n=90; time 2: n=83; time 3: n=80; and time 4: n=79.

**Figure 3:**
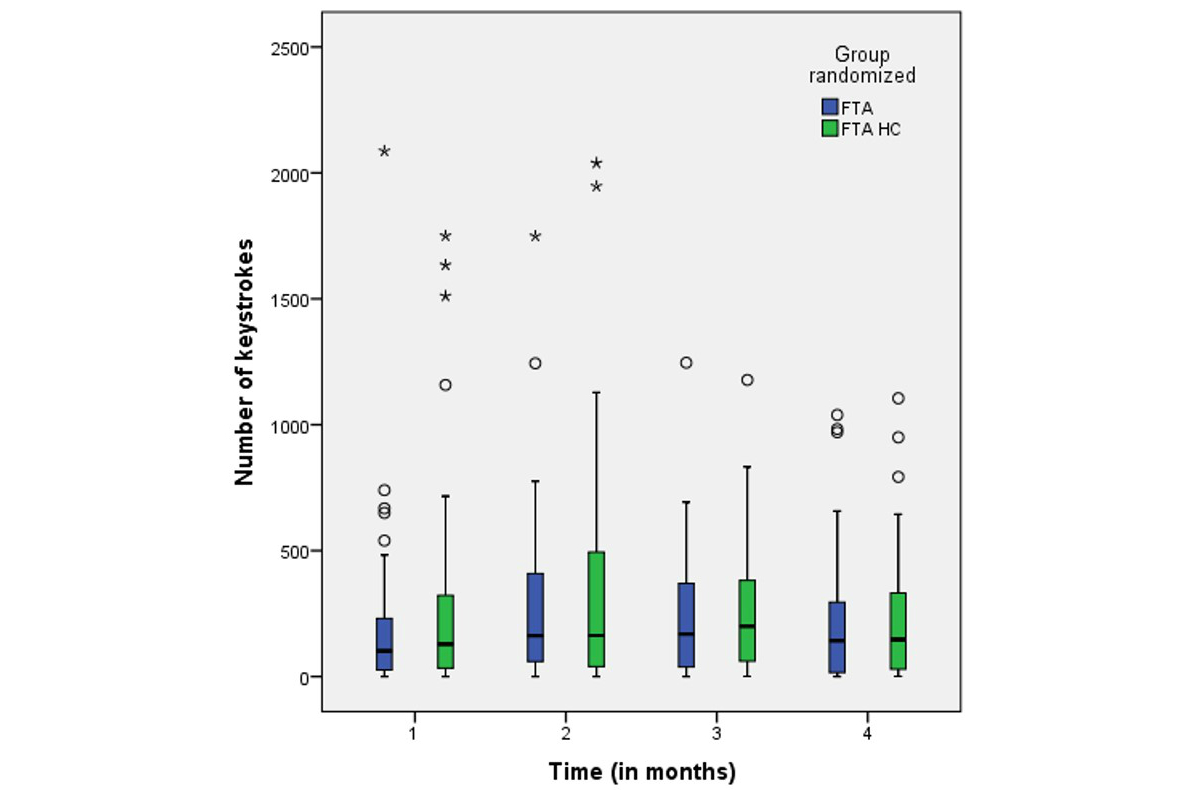
Number of keystrokes during the first 4 months for the 2 intervention groups: Few Touch Application (FTA) and FTA with health counseling (HC). Time 1 (baseline): n=90; time 2: n=83; time 3: n=80; and time 4: n=79.

The trend in the use of the app was not particularly different between the 2 intervention groups regarding either the number of blood glucose measurements (Figure 2) or number of keystrokes (Figure 3). The degree of use was lowest during the first month; it increased slightly during the second month and remained at about the same level during the third and fourth months.

**Table 5:** Changes in 2 heiQ domains from baseline to 4 months.

Domain and group		Baseline		4 months		Mean change	
		n	Mean (95% CI)	n	Mean (95% CI)	n	Mean (95% CI)
**Skills and technique acquisition**							
	FTA	51	2.95 (2.82, 3.07)	40	2.98 (2.81, 3.15)	40	0.02 (–0.12, 0.16)
	FTA with health counseling	50	2.87 (2.75, 2.99)	41	3.04 (2.90, 3.19)	41	0.17 (0.04, 0.29)
	Control	50	2.92 (2.83, 3.02)	43	2.92 (2.76, 3.07)	43	–0.04 (–0.18, 0.09)
**Health service navigation**							
	FTA	51	3.14 (3.00, 3.28)	40	3.21 (3.04, 3.37)	40	0.02 (–0.10, 0.14)
	FTA with health counseling	50	3.08 (2.95, 3.20)	41	3.27 (3.11, 3.42)	41	0.22 (0.07, 0.37)
	Control	50	3.13 (2.98, 3.27)	43	3.20 (3.05, 3.35)	43	0.00 (–0.11, 0.12)

**Table 6:** Linear regression analysis with crude and adjusted values for HbA_1c_ and heiQ domains from baseline to 4-month follow-up.

Group			n	Unadjusted		Adjusted^a^	
				Estimated β (95% CI)	*P*	Estimated β (95% CI)	*P*
**HbA**_**1c**_							
	FTA		40	.02 (–.40, .44)	.91	.03 (–.40, .46)	.90
	FTA with health counseling		39	.18 (–.24, .60)	.40	.16 (–.27, .58)	.47
	Control (ref)		39				
**heiQ domains**							
	**Skills and technique acquisition**						
		FTA	40	–0.21 (–0.39, –0.03)	.02	–0.22 (–0.40, –0.03)	.02
		FTA with health counseling	41	–0.14 (–0.33, 0.04)	.13	–0.15 (–0.34, 0.03)	.11
		Control (ref)	43				
	**Health service navigation**						
		FTA	40	–0.21 (–0.39, –0.04)	.02	–0.23 (–0.41, –0.05)	.01
		FTA with health counseling	41	–0.20 (–0.38, –0.02)	.03	–0.19 (–0.37, –0.01)	.04
		Control (ref)	43				

^a^ Adjusted for age, gender, and education.

### Adverse Events

No adverse events or important unintended effects were reported. Two persons died during the study, but these events were not related to the intervention or the study overall.

## Discussion

We found no significant changes between groups for the primary outcome measure HbA_1c_, although there were declines in the control group and in the intervention groups from baseline to 4-month follow-up. However, to the best of our knowledge, this is the first study to describe an effect of an electronic diabetes diary (FTA) in persons with type 2 diabetes in terms of their self-management and confidence in their capacity for health service navigation which may indicate an improved understanding of how to access health care to meet their needs. In addition, we found that the participants developed skills and technique acquisition, indicating that they improved their skills in relieving symptoms and gaining better health (according to heiQ) [13]. The FTA with or without health counseling from the diabetes specialist nurse appears to be a supporting tool that improved perceived self-management and it may have mitigated the burden caused by the illness.

The reasons for the lack of effect on the primary outcome of HbA_1c_ between groups are not clear, but several explanations are possible.

First, the HbA_1c_ level declined in all groups, thus the FTA intervention with or without health counseling may not be sufficiently effective, at least in the short term, to encourage a sufficient change in lifestyle to cause a further decrease in HbA_1c_. It is also reasonable to question what outcome measures could be used to better judge the effectiveness of self-management interventions and to evaluate behavior change [40,44]. The participants in our study had a mean diabetes duration of approximately 10 years and approximately 60% were obese, 50% reported 2 or more comorbidities, and only 7% did not receive glucose-lowering medication. In total, 31% of the participants were treated with both oral medication and injections, indicating that they had serious disease, which makes it difficult to reduce HbA_1c_ with a low-intensity lifestyle intervention. Thus, a higher intensity intervention may be required that considers the complexity of chronic conditions, whereas the low-intensity intervention used in our study provided less support and less frequent contacts with the health care providers [11,26]. However, after we adjusted for BMI, comorbidities, and medication, we found no indications that the effect differed between those with high and low BMI or disease burdens. Irrespective of these findings, one may nevertheless speculate whether a low-intensity intervention is appropriate for people who have been living with diabetes for a long time and if it is realistic to think that lifestyle changes can result in improved self-management and weight reduction. More recently, research has indicated that contact in clinical practice through telemedicine should be increased over time [18]. Many patients need closer support with structured interventions to help them attain the goals that they chose [15].

The FTA intervention could also have been too time-consuming because it required daily recordings of blood glucose, diet, and physical activity, and even more for the group that received additional health counseling. However, the app was accessed via the smartphone distributed in the project and it could be used as their own and when convenient. Another aspect of interest in this intervention is the health psychology models used in the health counseling and the proper use of theories in mHealth in general. Different directions within health psychology may also suit different people. More research within this area is needed. A transdisciplinary research approach is necessary in this matter and this is an area in which technology and psychology have to cooperate closer in the future.

Blinding of participants and health care personnel was not possible and the decline in the HbA_1c_ level in all groups, including the controls, may be attributed to the Hawthorne effect, particularly the attention the participants received when joining the study, which may have increased their self-confidence with respect to their diabetes management. They may also have received special attention from their general practitioners because “their patients” were included in a lifestyle intervention with modern technology [45]. Furthermore, according to the study design, a run-in period prior to randomization could have helped to stabilize the HbA_1c_ level before the study started, but we lacked the resources and the time for this additional process. However, a run-in period could also have led to increased dropouts, which in turn could have threatened the external validity if only participants that were highly motivated by a telemedicine intervention were randomized. In addition, expectations about the project and the possible intervention could have increased during a run-in period; thus, the participants who were disappointed about not receiving the expected intervention might have caused further dropouts and threatened a successful randomization due to dropouts from causes other than usual [46-49]. To address this challenge, a stepped wedge trial design, in which all participants received the intervention gradually could have compensated for the dilemma of withholding the intervention and the related Hawthorne effect. However, the design would then have been expensive because of the length of the intervention and the demands of collecting data [50]. More research is needed to optimize intervention-based research designs for patients with diabetes, as discussed previously [51].

It was also interesting that several participants wanted to attend the study although they were not eligible according to the eligibility criterion of HbA_1c_ ≥7.1%, as indicated in the flow diagram. This suggests that even though they were within their recommended treatment goals, they felt the need for professional support to facilitate a lifestyle change in addition to their use of medication. This should be taken into consideration when deciding the inclusion criteria and using HbA_1c_ as a primary outcome in future research.

With respect to the self-management measures, we found that the participants in both intervention groups reported significantly better scores for the heiQ health service navigation domain, whereas the intervention group that received FTA also reported significantly better scores in the skill and technique acquisition domain. Increased skill and technique acquisition may indicate an increased ability to reduce symptoms and manage health challenges, including the use of management devices. Furthermore, the health service navigation domain indicates that communication with health personnel is improved and that the communication is more specific to the patient’s own health needs [13]. It appears that the participants’ self-management skills and ability to make contact with health personnel increased during the intervention, whereas typical well-being domains, such as emotional well-being, social integration and support, and positive and active engagement in life, remain unchanged after 4 months. These results extend the findings of Nolte [44] by confirming that self-management courses appear to improve these skills in patients with chronic diseases.

The strengths of this study are that it was an RCT with 3 arms of equal size and few differences between groups and equal dropouts. The control group provided an opportunity to compare the standard treatment with a mobile health intervention based on theory. According to the power calculation based on the HbA_1c_, the sample size was acceptable and it provided sufficient support for the primary outcome, but the sample and subgroups were still small and they did not allow subgroup analysis as desired.

Another limitation is that the participants and their general practitioners were not blinded, indicating there was greater opportunity for the participants to influence the results. For example, the control group could have used similar apps. However, the app was meant to be shared with others, such as health care personnel, and the participants were expected to communicate and clarify their needs. This could have affected the intervention groups, but also the controls.

Finally, technology is developing rapidly. When the inclusion period was extended to recruit sufficient participants, the smartphone used was gradually lagging behind the latest smartphone software released onto the market. We found that an immediate transfer of the app to another mobile software system was too demanding, despite the risk of reduced interest in the app. The use of new software could have changed the intervention because the participants would also have been able to use the smartphone for calls and a more user-friendly phone could have changed perceptions of the app’s accessibility and usability.

The significant differences between the randomized groups were slightly uneven with respect to the distribution of rheumatic diseases and depression. Both of these diseases and their treatments can affect self-management and influence the HbA_1c_ levels. However, the estimates did not change after adjusting for these variables. As mentioned earlier, the randomization procedure was generally successful with 3 equal groups at baseline and the dropouts were distributed almost equally among the groups.

The use of the FTA diabetes diary with or without additional health counseling improved self-management in terms of the ability to navigate health services and the skills required to reduce symptoms. The app and the health counseling did not help to reduce the HbA_1c_ levels of the participants in the intervention groups compared with those who received usual care.

## Abbreviations

BMI: body mass index
CIP: Competitiveness and Innovation Framework Programme
CONSORT: Consolidated Standards of Reporting Trials
FTA: Few Touch Application
HbA1c: glycated hemoglobin A1c
heiQ: Health Education Impact Questionnaire
MAST: Model for the Assessment of Telemedicine
RCT: randomized controlled trial
RENEWING HEALTH: REgioNs of Europe WorkING together for HEALTH
USB: universal serial bus
